# Exploring the application and future outlook of Artificial intelligence in pancreatic cancer

**DOI:** 10.3389/fonc.2024.1345810

**Published:** 2024-02-21

**Authors:** Guohua Zhao, Xi Chen, Mengying Zhu, Yang Liu, Yue Wang

**Affiliations:** ^1^ Department of General Surgery, Cancer Hospital of China Medical University, Liaoning Cancer Hospital & Institute, Liaoning, China; ^2^ Department of Clinical integration of traditional Chinese and Western medicine, Liaoning University of Traditional Chinese Medicine, Liaoning, China; ^3^ Department of Ophthalmology, First Hospital of China Medical University, Liaoning, China

**Keywords:** pancreatic cancer, Artificial intelligence, early screening, personalized treatment, management

## Abstract

Pancreatic cancer, an exceptionally malignant tumor of the digestive system, presents a challenge due to its lack of typical early symptoms and highly invasive nature. The majority of pancreatic cancer patients are diagnosed when curative surgical resection is no longer possible, resulting in a poor overall prognosis. In recent years, the rapid progress of Artificial intelligence (AI) in the medical field has led to the extensive utilization of machine learning and deep learning as the prevailing approaches. Various models based on AI technology have been employed in the early screening, diagnosis, treatment, and prognostic prediction of pancreatic cancer patients. Furthermore, the development and application of three-dimensional visualization and augmented reality navigation techniques have also found their way into pancreatic cancer surgery. This article provides a concise summary of the current state of AI technology in pancreatic cancer and offers a promising outlook for its future applications.

## 1 Introduction

Pancreatic carcinoma represents a prevalent malignancy of the digestive system, characterized by elusive prodromal symptoms, heightened invasiveness, and an elevated propensity for postoperative metastasis and relapse. As delineated by the International Agency for Research on Cancer, pancreatic cancer assumes the fourteenth position amidst the 36 most prevalent malignant neoplasms in terms of incidence, and ranks seventh in mortality rates (1). An additional epidemiological analysis revealed a staggering 60,430 newly diagnosed cases of pancreatic cancer, resulting in 48,220 fatalities within the United States during the year 2021 (2). Projections suggest that by 2030, pancreatic cancer could ascend to become the second leading cause of cancer-related mortality in the United States (3). The sentence is clear and properly structured. The current diagnostic paradigm for pancreatic cancer primarily hinges upon imaging modalities such as CT and MRI, supplemented by the judicious use of PET-CT for comprehensive assessment when warranted. Notably, endoscopic ultrasonography (EUS) and EUS-guided fine needle biopsy assume pivotal roles in the diagnosis and staging of pancreatic cancer, albeit their diagnostic precision is subject to multifarious technical considerations. While radical surgery remains the cornerstone of pancreatic carcinoma management (4), neoadjuvant therapy has emerged as a burgeoning area of investigation (5). However, the absence of a standardized framework for assessing its efficacy poses a significant challenge. Furthermore, the prognosis following pancreatic cancer resection remains fraught with a substantial risk of tumor recurrence, thereby necessitating vigilant surveillance through regular follow-up regimens.

The term “Artificial intelligence (AI)” was first coined in 1956, with a focus on using machines to imitate human learning and cognitive abilities. Machine learning, a practical application of AI, employs diverse algorithms such as decision trees, random forests, artificial neural networks, support vector machines, logistic regression, Bayesian methods, K-nearest neighbors, among others. One such method, deep learning, is classified under the category of artificial neural networks (6) and has exhibited exceptional performance in image processing, notably through convolutional neural networks. By accurately and objectively identifying characteristic values of images based on standardized decision-making protocols, deep learning can comprehensively analyze statistical relationships between these values and associated outcomes. AI-based radiomics can thus achieve precise diagnosis of pancreatic cancer, while deep learning can establish early high-risk prediction models for pancreatic cancer and postoperative recurrence risk prediction models, facilitating early screening and assisting in the management of complications after pancreatic cancer surgery.

## 2 AI-assisted early screening and risk prediction of pancreatic cancer

By harnessing foundational data encompassing precancerous lesions, population-level health parameters, and a spectrum of biological markers pertinent to pancreatic cancer, AI holds promise in constructing predictive models to gauge the propensity for pancreatic cancer incidence, thereby enabling early detection and intervention. Intraductal papillary mucinous neoplasm (IPMN) serves as a precursor lesion in the development of pancreatic cancer (7). To explore IPMN, a study (8) curated an extensive compendium of 3,970 endoscopic ultrasound (EUS) images sourced from histopathologically validated IPMN patients, serving as inputs for sophisticated deep learning algorithms. Introducing the concept of the AI value, a continuous variable spanning 0 to 1, as well as the AI malignancy probability, denoting the mean AI value per patient, the researchers discerned markedly elevated average AI values in malignant IPMN vis-à-vis benign instances (0.808 vs. 0.104, P<0.01). Importantly, the model showcased commendable predictive prowess, exemplified by an impressive area under the curve (AUC) of 0.98 for the receiver operating characteristic curve of AI malignancy probability, thus validating its potential in prognosticating the transformation of IPMN into malignant tumors. Notably, the AI-based diagnosis exhibited superior sensitivity, specificity, and accuracy (95.7%, 92.6%, and 94.0% respectively) compared to physicians’ diagnostic accuracy (56.0%).

Constituting a reservoir of cumulative healthcare data, longitudinal electronic health records have emerged as a pivotal asset for researchers endeavoring to construct predictive models targeting medical prognostication. Recent investigations spearheaded (9–11) have honed in on the development of predictive frameworks tailored to unearthing high-risk subcohorts vulnerable to pancreatic cancer within the diabetic patient cohort. Particularly salient is the work (9), that derived a prognostic schema from a cohort of newly diagnosed diabetes patients, synthesizing key determinants encompassing age at diabetes onset, body mass index, and glycemic fluctuations. This intricately woven algorithm furnished predictive scores adept at forecasting the incipient trajectory toward pancreatic neoplasms within a triennial window. Notably, patients scoring 3 or above evinced a diagnostic sensitivity and specificity of 80% for pancreatic carcinoma (AUC=0.87), marking a 4.4-fold escalation in pancreatic cancer incidence vis-à-vis their diabetic counterparts. Meanwhile, another study (10) leveraged both logistic regression and artificial neural network methodologies to craft predictive architectures pertinent to type 2 diabetes patients in Taiwan, marshaling parameters inclusive of age, antidiabetic pharmaceutical usage, and comorbid ailments as prospective risk determinants for pancreatic cancer. Intriguingly, the logistic regression model emerged as the more discerning performer, boasting an AUC of 0.727. In a similar vein, Blyuss et al. (12) developed a novel pancreatic cancer patient risk scoring system (PancRisk) predicated on urinary biomarkers. The team measured three urine biomarkers (LYVE1, REC1B, TFF1) in 199 pancreatic ductal adenocarcinoma patients and 180 healthy individuals and applied machine learning algorithms to analyze and compare the datasets. The resulting logistic regression model demonstrated remarkable diagnostic power with an AUC of 0.94. When combined with the established tumor marker CA19-9, the model achieved a diagnostic specificity and sensitivity of 96%.

## 3 AI-assisted diagnosis of pancreatic cancer

Diagnosing pancreatic cancer is a multifaceted process that typically involves clinical manifestations, high-risk factors, serum tumor markers, and imaging techniques such as endoscopic ultrasound (EUS). However, imaging examination remains the most crucial approach for clinical diagnosis of pancreatic cancer, with contrast-enhanced CT and MRI being the standard options (13). In recent years, deep learning models have emerged as a promising tool to aid pancreatic cancer diagnosis. Zhejiang University, for example, developed a deep learning model trained on 319 patients’ abdominal contrast-enhanced CT images that could provide pancreatic tumor diagnosis suggestions based on original abdominal CT images without preprocessing. Impressively, this model achieved an AUC of 0.871 and an F1 score of 88.5%, with an average diagnostic accuracy of 82.7% for all tumor types. Moreover, the model demonstrated exceptional accuracy in distinguishing IPMN and pancreatic ductal adenocarcinoma, reaching 100% and 87.6%, respectively (14). Additionally, a study from China (15) developed a convolutional neural network model trained on 7245 CT images from 222 pathologically confirmed pancreatic cancer patients and 190 normal pancreatic patients. The model was trained to differentiate between two categories (with or without pancreatic cancer) and three categories (no cancer, tumor in the body/tail of the pancreas, tumor in the head/neck of the pancreas), demonstrating remarkable accuracy in diagnosing plain scan images (95.47%) with high sensitivity (91.58%) and specificity (98.27%). Notably, the three-category model proved particularly adept at diagnosing tumors in the head/neck of the pancreas using arterial phase images.

Endoscopic ultrasound (EUS) is an indispensable tool for diagnosing pancreatic tumors and chronic pancreatitis (13). With the advent of AI, EUS images’ diagnostic efficiency has been remarkably improved. To address the challenge of distinguishing autoimmune pancreatitis (AIP) from pancreatic ductal adenocarcinoma, chronic pancreatitis, and normal pancreas, Marya et al. (16) developed a convolutional neural network model based on EUS images. The model was trained using static images and videos from EUS examinations of patients. Impressively, the model achieved high sensitivity and specificity rates for differentiating AIP from pancreatic ductal adenocarcinoma (90% and 93%, respectively), normal pancreas (99% and 98%, respectively), and chronic pancreatitis (94% and 71%, respectively). Moreover, another research (17) implemented age grouping into the AI model trained on EUS images and conducted stratified analysis by dividing patients into three age groups (<40 years old, 40-60 years old, and >60 years old). The results showed that the grouped model outperformed the ungrouped one in terms of classification accuracy, sensitivity, and specificity, with rates ranging from 88.5% to 94.1%. Notably, the study highlights the importance of age grouping in enhancing the diagnostic efficiency of AI models for EUS images.

Additionally, substantial strides have been made in AI-driven research concerning tissue pathology slices and tumor biology markers. Notably, unsupervised learning methodologies have demonstrated efficacy in the identification of specific tumor markers linked to pancreatic cancer (18), offering a novel approach to screening potential markers with clinical relevance. Furthermore, the pursuit of developing a sophisticated model capable of precisely identifying and autonomously segmenting pancreatic tumors stands as a critical frontier in medical research, holding great promise for advancing diagnostic capabilities and refining treatment modalities for pancreatic malignancies. These developments underscore the transformative potential of AI in reshaping the landscape of pancreatic cancer research and clinical practice.

## 4 Application of AI in the surgical treatment of pancreatic cancer

At present, radical resection surgery represents the cornerstone of curative strategies for pancreatic cancer. A seminal report published in 2006 chronicled a remarkable series of 1,000 consecutive pancreatico-duodenectomies performed by an esteemed surgeon at Johns Hopkins Hospital between 1969 and 2003 (19). Over this period, the frequency of these surgeries exhibited a steady rise, with only three cases documented prior to 1980. Remarkably, the median operating time decreased from 8.8 hours in the 1970s to 5.5 hours in the 2000s, yielding a strikingly low mortality rate of merely 1% within 30 days or during hospitalization. Akin to these findings, an extensive analysis encompassing 2,050 operations conducted at Massachusetts General Hospital between 1941 and 2011 further underscored the progressive improvements achieved in surgical management (20). Nevertheless, the advent and application of neoadjuvant therapy hold immense promise in broadening the population eligible for radical resection surgery and fostering improved prognoses. Notably, AI has emerged as a potent tool in the realm of neoadjuvant therapy for pancreatic cancer. A study from Netherlands conducted an insightful investigation to assess the efficacy of neoadjuvant therapy, employing histological examinations of surgical specimens following neoadjuvant chemotherapy (21). By employing digital processing techniques on HE-stained sections from 64 pancreatic cancer patients, they meticulously delineated three distinct categories (tumor, normal duct, and residual epithelium), effectively training a tumor segmentation model with an average F1 score of 0.86. Similarly, another study from USA utilized machine learning approaches to compare enhanced CT images before and after neoadjuvant therapy, successfully identifying and extracting treatment-related image features, culminating in the establishment of a prediction model boasting an impressive AUC of 0.94 (22). These seminal studies unequivocally affirm the feasibility of harnessing AI to evaluate the outcomes of neoadjuvant therapy in pancreatic cancer. By objectively assessing the response to neoadjuvant therapy, AI holds immense potential in guiding the selection of optimal neoadjuvant therapy regimens, thereby optimizing surgical interventions. Furthermore, researchers have made noteworthy strides in leveraging deep neural networks to precisely locate and even track pancreatic tumors without relying on internal markers (23). Additionally, they have pioneered automated segmentation methods for accurately delineating organ-threatening contours, providing invaluable guidance for radiotherapy planning (24). Deep learning techniques have also emerged as a valuable asset in the treatment planning of stereotactic radiotherapy for pancreatic cancer, enabling accurate predictions of radiation dose distribution (25).

AI has emerged as a potent tool in the field of surgery, particularly in the domain of three-dimensional reconstruction and visualization. A study form China published a seminal study demonstrating the high accuracy, sensitivity, and specificity of three-dimensional reconstruction in assessing pancreatic cancer (26). Collaborating with this team in 2019, we harnessed three-dimensional visualization technology to observe the location, size, and adjacency to surrounding organs of pancreatic head tumors prior to pancreaticoduodenectomy, optimizing surgical plans and effectively reducing surgical time, intraoperative blood loss, and postoperative recovery time for patients (27). In a similar vein, a study from Japan used three-dimensional reconstruction before surgery to precisely determine the size and position of the main pancreatic duct and select the best anastomosis technique (28). Augmented reality navigation technology represents a promising surgical navigation technique that merges three-dimensional virtual images with real-time intraoperative conditions. Okamoto et al. conducted a rigorous evaluation of five patients who underwent augmented reality navigation-assisted pancreatic resection surgery, revealing strong agreement between the positions of various organs in surface-stained images and their actual positions (29). Additionally, Volonte et al. applied augmented reality navigation technology to laparoscopic distal pancreatectomy, projecting nodules in the tail of the pancreas onto the patient’s body, enhancing anatomical understanding and localization for physicians (30). Moreover, Tang et al. even employed augmented reality software on smartphones to overlay reconstructed three-dimensional images onto the surgical area displayed on the phone screen, providing intermittent navigation assistance that helped identify the boundaries of pancreatic head cancer invasion and facilitate the removal of relevant blood vessels, ultimately achieving R0 resection for all surgical patients (31). The integration of AI into surgical practice holds great promise in improving patient outcomes through precise and personalized surgical interventions.

## 5 AI-assisted prediction and management of postoperative complications in pancreatic cancer patients

Pancreatic cancer surgery is often burdened by postoperative complications, including postoperative pancreatic fistula, bile fistula, postoperative bleeding, abdominal infection, and delayed gastric emptying (32, 33). The most common complication is pancreatic fistula, which can lead to serious complications such as abdominal infection and life-threatening bleeding. However, the current risk scoring system for pancreatic fistula only considers four factors, which has significant limitations (34). To address this issue, scholars in Korea developed an AI-driven postoperative pancreatic fistula risk prediction platform using random forest and neural network algorithms to analyze 38 variables from 1,769 patients who underwent pancreaticoduodenectomy (PD) from 2007 to 2016 (35). By combining neural networks with recursive feature elimination, the platform achieved a maximum AUC of 0.74, ultimately identifying 16 risk factors for postoperative pancreatic fistula, including pancreatic duct diameter, body mass index, preoperative serum albumin, lipase level, and age, among others. In addition, Skawran et al. used a gradient boosting tree model based on MRI radiomics to predict postoperative pancreatic fistula after PD, achieving a high AUC of 0.90 (36). Furthermore, Zhang et al. developed a predictive model for postoperative ICU admission with an AUC of 0.8 by employing a support vector machine model to analyze clinical features of patients with pancreatic ductal adenocarcinoma, revealing bilirubin, CA19-9, and preoperative albumin as associated factors for postoperative bleeding in patients (37). The use of AI in predicting postoperative complications in pancreatic cancer surgery holds great potential in improving patient outcomes and facilitating targeted treatment strategies.

## 6 AI prediction of prognosis in pancreatic cancer patients

The relationship between patient survival and recurrence in pancreatic cancer is of utmost importance, necessitating the identification of relevant factors contributing to recurrence. Lee et al. conducted a meticulous analysis using multicenter registry data to evaluate the probability of postoperative recurrence and ascertain its major prognostic factors in pancreatic cancer (38). By employing random forest and Cox proportional hazards models, disease-free survival was predicted in a large cohort of 4,846 patients. Remarkably, tumor size, tumor grade, TNM stage, T stage, and lymphovascular invasion emerged as key prognostic factors for postoperative disease-free survival based on their variable importance. The Cox model exhibited a higher mean C-index (0.7738) compared to the random forest model (0.6805), indicating its superior predictive ability. Additionally, Tong et al. (39) conducted a study involving 221 patients with unresectable pancreatic cancer, collecting data on 32 clinical parameters. They developed three artificial neural network models based on different sets of basic features (3, 7, and 32) to predict the 8-month survival rate of patients. Impressively, all three artificial neural network models exhibited favorable performance, surpassing the corresponding logistic regression models in terms of AUC values (0.811 vs. 0.680, 0.844 vs. 0.722, 0.921 vs. 0.849, all P < 0.05). These findings emphasize the potential of artificial neural networks in accurately predicting the survival rate of patients with unresectable pancreatic cancer.

## 7 Summary and outlook

In recent years, the rapid evolution of deep learning and AI has engendered burgeoning interest in their potential implications in the realm of pancreatic cancer. The application of AI technology has exhibited substantial promise in the realms of early screening, diagnosis, surgical interventions, and prognostic evaluations for pancreatic cancer, equipping clinicians with more precise and expeditious decision-making tools, consequently ameliorating treatment outcomes and enhancing patients’ survival rates (40). Nevertheless, notwithstanding the manifold affirmative prospects for the integration of AI in the domain of pancreatic cancer, certain constraints are inevitably encountered. Firstly, the interpretability conundrum of deep learning models utilized in pancreatic cancer screening, diagnosis, surgery, and prognostication frequently lacks transparency, impeding comprehension and engendering skepticism. Consequently, dedicated research endeavors are imperative to heighten interpretability and foster a more transparent decision-making process. Secondly, the generalization capacity of models across heterogeneous datasets is a significant concern. While deep learning models for pancreatic cancer developed on single-center datasets may demonstrate disparate accuracies when transposed to alternative medical facilities, enhancing generalization capacity assumes paramount importance in ensuring consistent performance across diverse clinical settings (41). Moreover, the limited sample size inherent in rare disease models such as pancreatic cancer poses a formidable obstacle to effective training and validation, culminating in erratic performance. Innovative methodologies including cross-center collaboration and synthetic sample generation warrant exploration to surmount this challenge and bolster reliability (42). Finally, the normative intricacies surrounding the utilization of AI in pancreatic cancer diagnosis and treatment necessitate the establishment of ethical benchmarks and standards to safeguard patient confidentiality and data integrity. Research initiatives should be concentrated on formulating pertinent protocols and mechanisms for data dissemination, while upholding the sanctity of patient rights and privacy (43). In conclusion, despite the hurdles associated with interpretability, generalization, sample size, and ethical considerations, the potential dividends of deep learning and AI in pancreatic cancer research are profound. Future pursuits should revolve around the amalgamation of multi-omics data analysis to devise personalized treatment regimens tailored to individual patients, ultimately augmenting therapeutic efficacy and survival rates (44, 45). Synergistic collaborations between clinicians and researchers are indispensable in effectuating the seamless integration of these technologies into clinical practice.

## Author contributions

GZ: Investigation, Writing – review & editing. XC: Writing – original draft. MZ: Investigation, Validation, Writing – review & editing. YL: Investigation, Writing – review & editing. YW: Conceptualization, Writing – review & editing.
